# Wet healing and Chinese medicine cupping can be used to form collateral circulation in diabetic foot artery occlusion: a case report

**DOI:** 10.3389/fendo.2025.1583647

**Published:** 2025-09-12

**Authors:** Shengmei Wu, Qianqian Peng, Chunlei Tian, Junfeng Hu, Xiandi Wu, Lili Zhang, Jin Zhang

**Affiliations:** ^1^ China Three Gorges University, Yichang, China; ^2^ The First College of Clinical Medical Science, China Three Gorges University, Yichang Central People's Hospital, Yichang, China; ^3^ People’s Hospital of Zhijiang, Yichang, China

**Keywords:** diabetic foot ulcer, traditional Chinese cupping, wet healing, local blood circulation, wound healing

## Abstract

This report describes a diabetic patient with a left little toe ulcer who was treated with Wet healing combined with traditional Chinese cupping. This approach successfully improved local blood circulation and facilitated wound healing. This treatment modality provides a new perspective for the comprehensive management of diabetic foot ulcers.

## 1 Introduction

This report describes a diabetic patient with a left little toe ulcer who was treated with combined with traditional Chinese cupping wet therapy. This approach successfully improved local blood circulation and facilitated wound healing. This treatment modality provides a new perspective for the comprehensive management of diabetic foot ulcers.

## 2 Background

Diabetic foot ulcer (DFU) is a common chronic wound and complication of diabetes (1). Diabetic ulcers primarily affect the foot, affecting peripheral nerves, microcirculation, small arteries, and other structures. Many patients with diabetes suffer from DFU, which is prone to co-infection. Studies have indicated that between 4% and 10% of people suffer from diabetic foot worldwide, and this number may increase with the rising prevalence of diabetes (2). 2023 International Working Group on Diabetic Foot recommendations, the current treatment methods of diabetic foot include hyperbaric oxygen therapy, negative pressure drainage, and growth factors ^[0]^. Negative pressure cupping in traditional Chinese medicine is one of the core external treatment methods in traditional Chinese medicine. Through the negative pressure inside the cup, it adheres to the skin to produce biomechanical stimulation, thereby increasing local microcirculation perfusion and improving local blood circulation (3–5). The concept of wet healing is that keeping the wound in a moderately moist environment is more conducive to wound healing than the traditional dry and scabbed environment (6). Hydrogel dressings are widely utilized in the treatment of diabetic foot ulcers (7). The intrinsic three-dimensional (3D) porous structure of hydrogels closely resembles the extracellular matrix, which makes them particularly suitable for the targeted delivery and controlled release of bioactive agents to the wound site. Moreover, hydrogels are fully water-soluble and capable of rapidly responding to various local microenvironmental stimuli, such as changes in temperature, pressure, pH, or the presence of antigens (8). The principle of negative pressure vacuum cupping is to actively discharge the air in the tank through the suction device, thereby forming a negative pressure environment below atmospheric pressure. The pressure difference between inside and outside of the tank makes the tank adsorbed on the skin, resulting in local tissue lifting and physiological stimulation (9). A negative pressure in the wound bed can effectively remove fluid, which facilitates granulation tissue formation and helps the approximation of the wound edges. Wu et al. used negative pressure vacuum cupping to treat deep pressure ulcers (10). The conceptual framework of this study is grounded in the pathological mechanisms underlying diabetic foot ulcers. We hypothesize that wet healing combined with cupping therapy can enhance collateral circulation reconstruction, thereby facilitating wound repair and symptom alleviation through improved local blood flow and a favorable healing microenvironment.

## 3 Case presentation

The patient was a 68-year-old man weighing 70 kg. He had diabetes for 26 years, and hypertension for 9 years. He took nifedipine orally to control blood pressure and insulin to control blood glucose. In February 2021, he felt cold and numbness in his lower limbs without an obvious cause, which was aggravated after cold exposure and exercise, accompanied by intermittent claudication. The claudication distance was nearly 500 meters, with no resting pain, no movement limitation, no chills and high fever, and no palpitations or shortness of breath. The distance of intermittent claudication of both lower limbs was shortened to 100 meters. There was a 1*1 cm ulcer on the little toe of the right foot, with a black scab and tenderness. He was treated in the local hospital, and color Doppler ultrasound of the lower limbs showed varying degrees of stenosis in bilateral anterior tibial arteries and posterior tibial arteries. Therefore, he was transferred to the superior hospital for treatment. The superior hospital performed lower extremity arteriography + balloon dilatation + stent formation on April 6, 2023, but angiography showed segmental occlusion of the three branches below the knee (anterior tibial artery, posterior tibial artery, and peroneal artery) when the 6F sheath tube was placed, and the peroneal artery still exhibited no reflow. Then, the secondary diabetic foot infection progressed. On May 22, 2023, the patient underwent debridement on the left side, amputation of the fifth toe, and debridement of metatarsal osteomyelitis. Debridement and drainage of the left foot were conducted on June 5, 2023. The patient elected for discharge against medical advice after declining recommended surgical reintervention (repeat debridement and possible re-amputation for persistent osteomyelitis).

**Figure 1:**
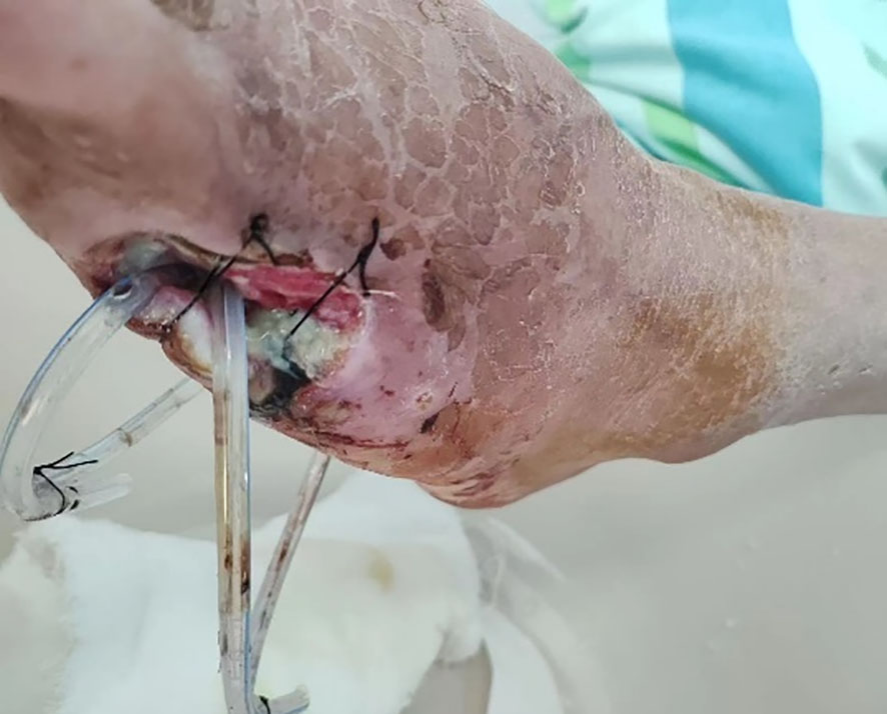
Instep.

**Figure 2:**
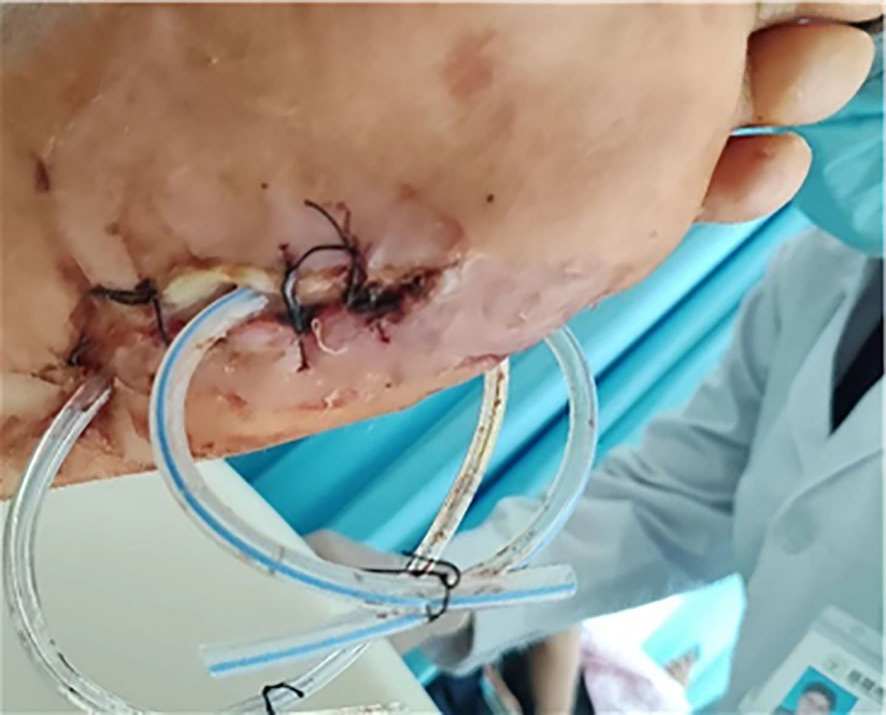
Sole of the foot.

For limb salvage treatment, the patient came to the wound care clinic of our hospital on June 25, 2023. During the initial consultation, the patient’s left foot was swollen, and there were two wounds (a 5 cm × 9 cm penetrating drainage wound and a 3 cm × 7 cm latent lacunar wound). The wound was yellow in more than 75% and black in less than 25%, with a large amount of purulent secretion and grade 4 odor. Tissue maceration, unremoved sutural residue, and focal necrosis were observed at the edge of the wound. The surrounding skin was red, swollen, macerated, and crusted, with the absence of the fifth toe. The patient was admitted to the hospital with the absence of the little finger of the left foot. A drainage tube was placed to drain the dorsum of the foot (**Figure 1**) and the sole of the foot (**Figure 2**).

### 3.1 Auxiliary test results

**Figure 3:**
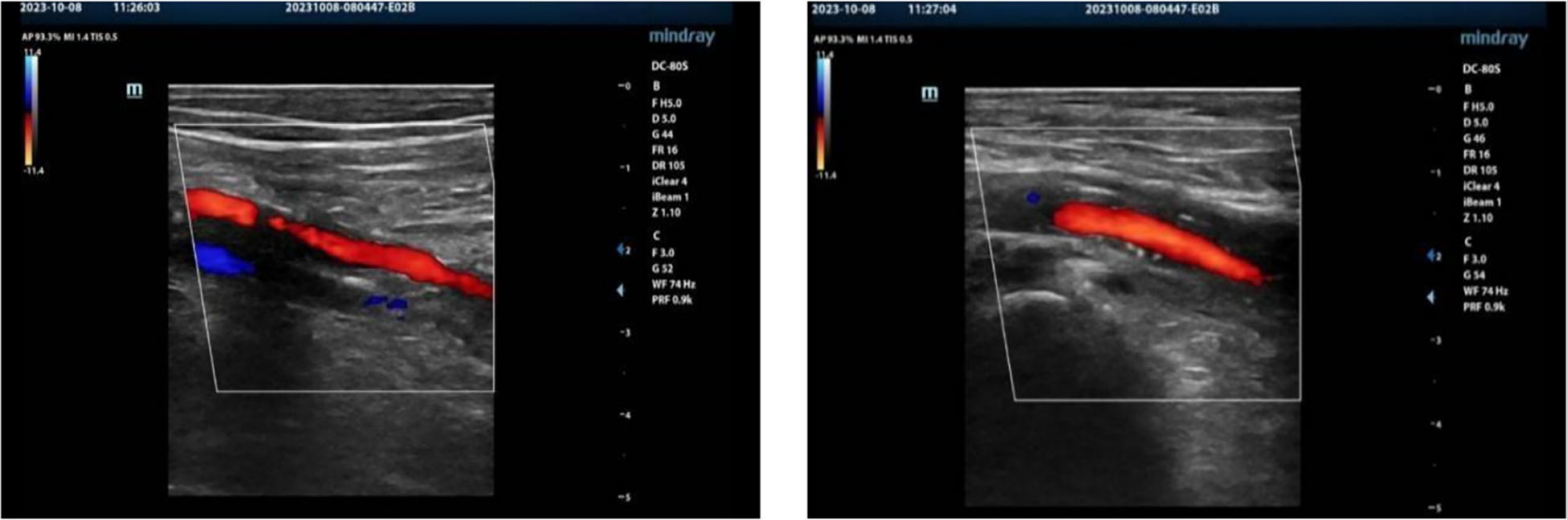
Ultrasound indicating stenosis of the lower segment of the left superficial femoral artery, deep femoral artery, popliteal artery, and posterior tibial artery. Closure of the left anterior tibial artery and the common peroneal artery. Normal blood flow in the veins of the left lower extremity.

**Figure 4:**
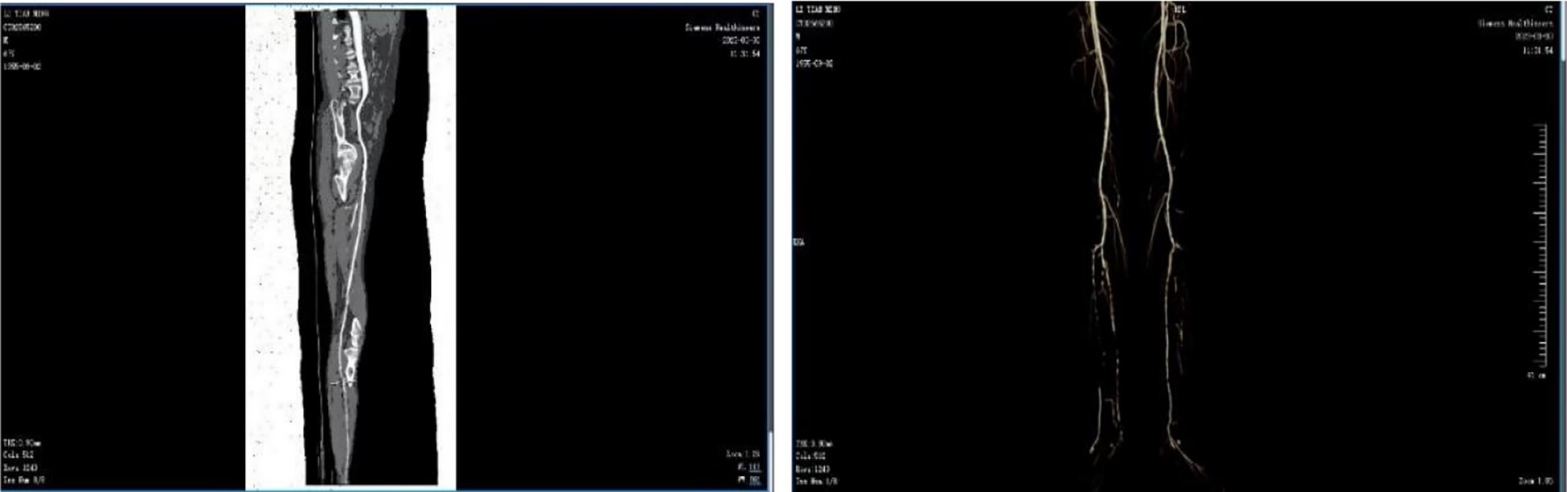
CTA showing irregular arterial walls with multiple patchy opacities and an irregular lumen. There was moderate to severe segmental stenosis in both the femoral and popliteal arteries, with severe stenosis in the proximal left profunda femoris. The anterior tibial, posterior tibial, and peroneal arteries were bilaterally occluded, with low-density opacities occurring in the posterior tibial arteries.

Ultrasound examination indicated that the patient had stenosis in the lower segment of the left femoral profunda artery, the popliteal artery, the tibialis posterior artery, and the posterior tibial artery (**Figure 3**). Of these, the peroneal artery and the left anterior tibial artery were already blocked. In addition, computed tomography angiography (CTA) showed that the patient’s proximal segment of the left deep artery exhibited severe stenosis (**Figure 4**). Blood test results also revealed abnormal values of calcium (1.99 mmol/L), potassium (3.34 mmol/L), albumin (ALB) (34.58 g/L), and total protein (TP) (60.35 g/L). Blood culture revealed that the patient had an Escherichia coli infection.

## 4 Cupping in conjunction with a wet healing procedure

### 4.1 Treatment measures included

#### 4.1.1 Blood glucose and blood pressure management: oral antidiabetic drugs

As recent studies have highlighted (11) that managing diabetic foot necessitates the assessment and maintenance of the patient’s overall health, with a particular focus on the role of cardiovascular homeostasis in regulating local healing. This principle is embodied in hypertensive patients with DFU. Blood pressure control and electrolyte balance together constitute “cyclin-metabolic dual-axis regulation”, which is a core systemic intervention. The patient received chewable acarbose 50 mg, 3 times a day with the first staple food and nifedipine 1 capsule, 2 times a day. Blood glucose was monitored regularly and adjusted continuously.

#### 4.1.2 Diet and nutritional support

Nutrition forms the cornerstone of wound treatment at this center. Professional nutritionists conduct comprehensive evaluations of all patients and develop individualized nutrition plans (including diabetes patients’ blood sugar management), which is a key feature for promoting healing. We regularly monitor indicators such as albumin to ensure that the nutritional status is conducive to recovery.

#### 4.1.3 Psychological care

The medical team actively listened and regularly communicated with patients, patiently listened to their concerns and feelings about the disease, treatment, and wound condition, and expressed empathy. Through regular photo comparisons, the patient was informed of positive changes in wound healing to affirm the patient’s cooperation with treatment. The medical team encouraged the patient and enhanced positive experiences and confidence. Small goals were set, and encouragement was offered. The rehabilitation process was divided into achievable small goals. Specific and sincere encouragement and affirmation were given for each achievement. Family members were encouraged to participate, provide emotional and life support to patients, and form a positive rehabilitation atmosphere. Blood pressure and blood glucose levels remained normal throughout the treatment.

### 4.2 The principle of local dressing change

**Figure 5:**
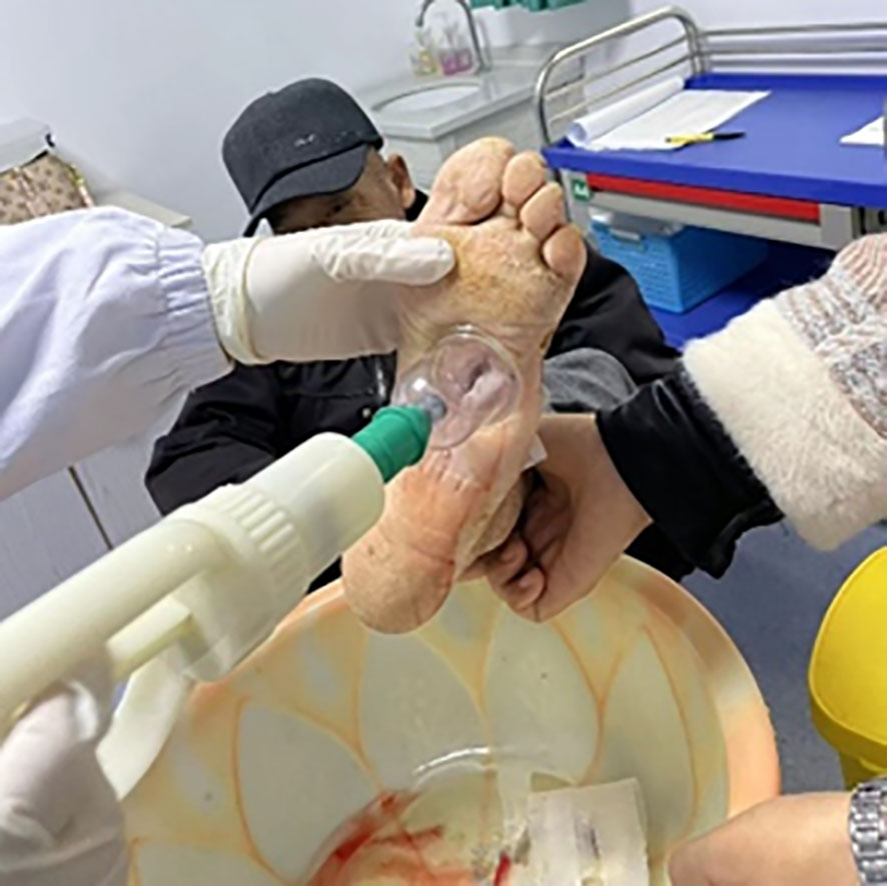
The cupping process.

1. Removing the drainage tube that affects wound healing and removing sutures.
2. Drug debridement combined with mechanical debridement to remove dead tissue:for the deep-seated sinus tract bone marrow abscess wounds, when performing deep debridement and dressing changes, a stepped debridement strategy is adopted: Firstly, through sharp debridement, the necrotic tissues within the sinus tract are prioritely removed to effectively destroy the bacterial biofilm, thereby controlling the infection source; subsequently, physiological saline pulse irrigation is used to maintain the physiological environment of the local tissues and simultaneously reduce the bacterial load to a safe threshold. For deep sinus tracts, the negative pressure cupping technique can promote capillary proliferation and improve the tissue oxygenation status, which may be related to the activation of the vascular endothelial growth factor pathway. In the later stage of treatment, lipid hydrocolloid silver sulfate dressings strips combined with hydrogels are used for wound filling, where the silver ion component provides broad-spectrum antibacterial protection, the hydrocolloid matrix helps regulate the protease activity of the wound, and the hydrogel maintains a moderately moist healing environment. This treatment plan effectively promotes the re-epithelialization process of deep sinus tracts by simultaneously clearing the biofilm, improving local perfusion, continuously controlling the infection, and optimizing the healing microenvironment.
3. Controlling the bacterial load, rationally using antibiotics and antibacterial dressings based on the results of blood culture.
4. Exudation management, using traditional dressings and new foam dressings to manage exudation.
5. Negative pressure therapy: innovative use of traditional Chinese medicine negative pressure vacuum cupping combined with external application of traditional Chinese medicine to facilitate wound healing (**Figure 5**).

### 4.3 Therapeutic process

#### 4.3.1 Dressing change decission

Treatment process based on wound drainage quantity and odor and treatment decisions are shown in **Table 1**.

**Table 1:** Dressing change process.

Assessment parameters	High exudate burden phase (daily change)	Moderate exudate burden phase (alternate-day change)	Low exudate burden phase (every 2-day change)
Exudate volume	> 3/4 of dressing area (Gauze pad)	> 1/2 - 3/4 of dressing area (foam dressing)	< 1/4 of dressing area (hydrocolloid/film)
Odor score	≥ 3 (foul/putrid)	2 (slight/mild odor)	0-1 (no odor)
DFU healing scale score	SINBAD ≥ 4/Wagner grade ≥ III	SINBAD 2-3/Wagner grade I-II	SINBAD ≤ 1/Wagner grade 0-I
Tissue type	> 50% slough/necrotic tissue	25-50% granulation tissue/mixed type	> 75% epithelialization

SINBAD Score: A 6-dimension prognostic scoring system for evaluating diabetic foot ulcers: *Site, Ischemia, Neuropathy, Bacterial infection, Area, Depth*: Wagner Classification: The Wagner-Meggitt Foot Ulcer Classification System grades ulcer severity based on *anatomic depth and infection severity*.

#### 4.3.2 Periodic cupping operation steps

##### 4.3.2.1 In the acute inflammatory phase (2023.6.25 7.19), the bandage was changed daily

1. cupping therapy was conducted using a vacuum negative pressure tank (- 125-300 mmHg) covering healthy skin wound around 5 cm, non-continuously for 3 minutes.
2. wound treatment: 0.5% saline vortex flush was used to sharply debride necrotic tissues. The wounds were coated with silver ion dressings and an anti-inflammatory plaster sterile cotton cushion. Then, a medical elastic bandage was used for fixation.

##### 4.3.2.2 Granulation growth period (2023.7.19 8.4) on alternate days

(1) cupping adjustment: to fan moving cupping (- 100-150 mmHg), discontinuously for 3 minutes to promote local blood flow; (2) medical pads drainage foam dressings + elastic bandage fixation covered more than half of the wound; Liquid more than medical cotton pad 3/4: silver ion dressing + foam dressing + elastic bandage fixation.

##### 4.3.2.3 Epithelial period (2023.8.4 11.15) two days change medicine

**Figure 6:**
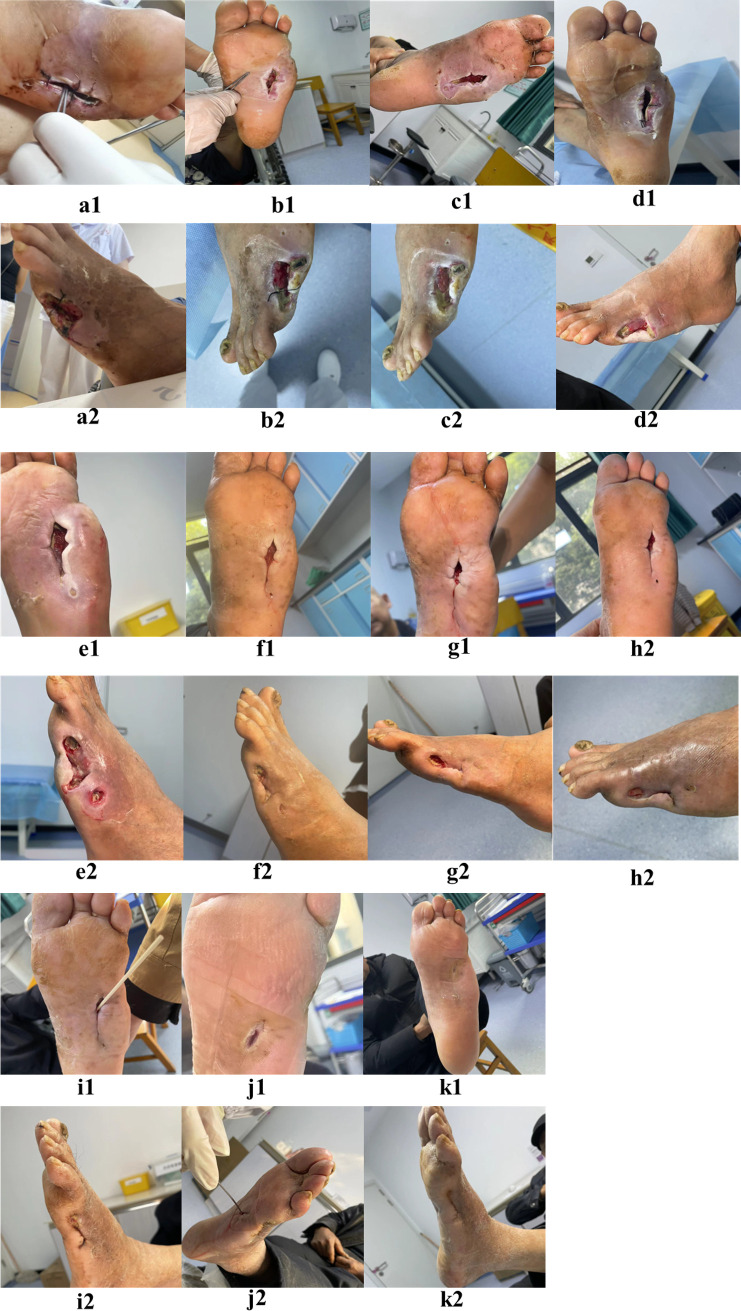
Images of dressing changes on June 26th, June 30th, July 3rd, July 21st, August 4th, August 30th, September 8th, September 22nd, October 23rd, January 5th, 2024, and January 29th, 2024 are labeled as **(a–k)** respectively.

(1) Indications for terminating cupping were as follows: wound shrink rate > 40% and no stealth; (2) new dressing:during the epithelialization dressing change, use hydrocolloid to protect the edges to prevent the wound from getting soaked.Some images of dressing change were selected monthly to assess the patient?s dressing change (**Figure 6**).

## 5 Results

**Figure 7:**
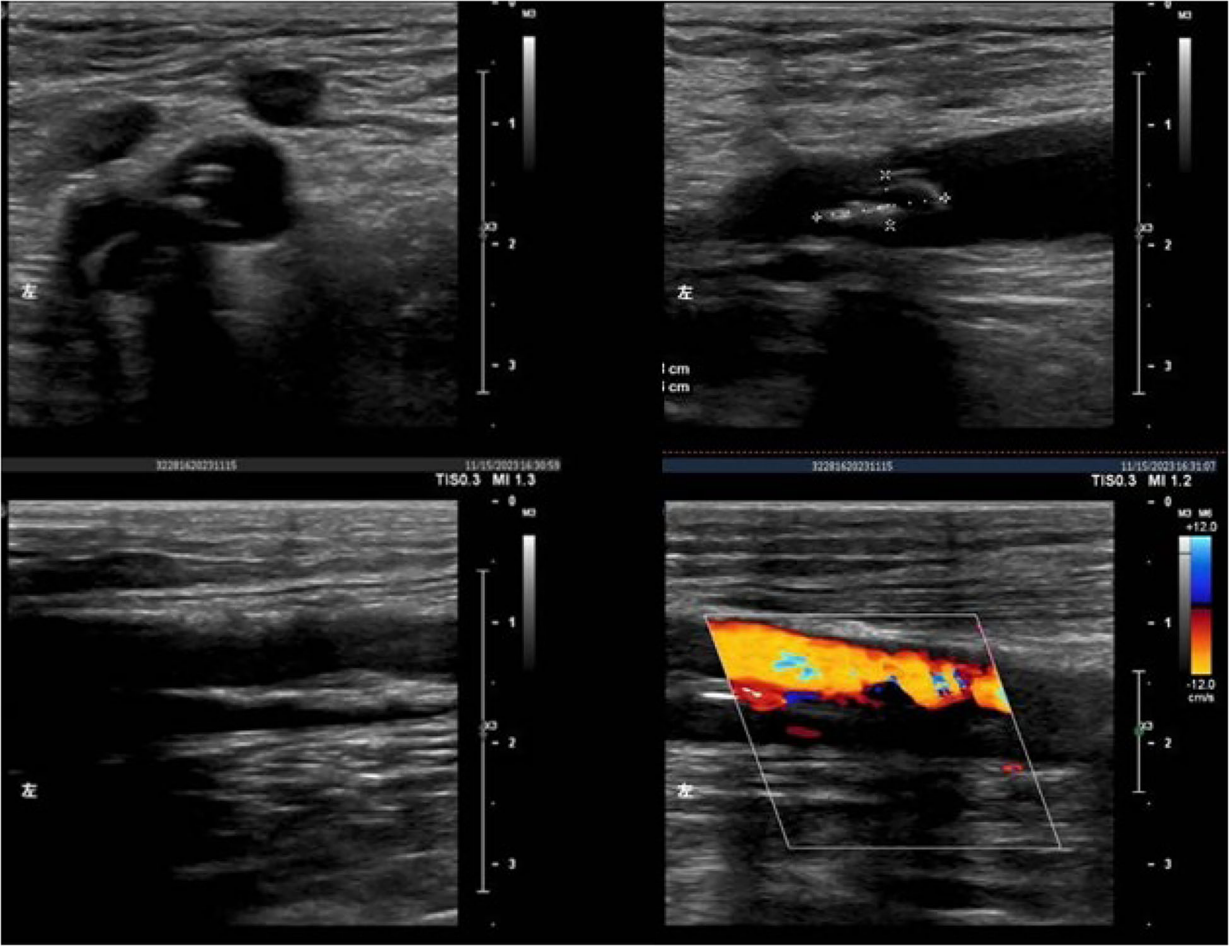
Ultrasound examination of the left lower extremity artery. The course of the left common femoral artery, the superficial femoral artery, the deep femoral artery, and the popliteal artery was normal. The entire vessel wall was not smooth. Multiple high-echo and low-echo plaques were observed in the left common femoral artery and superficial femoral arteries. One high-echo plaque was approximately 1.08 x 0.43 cm in size. Low-echo fillings were present in the left anterior tibial, posterior tibial arteries, and peroneal arteries.

K1 and K2 in **Figure 6** illustrate the wound’s healing after more than 140 days of treatment. Ultrasound showed that the patient’s left lower limb artery developed collateral blood vessels (**Figure 7**).

## 6 Discussion

DFU is the most prevalent, complex, and important health problem leading to disability in patients with diabetes (12). More than 50% of patients with DFU finally experience deep infection or gangrene, leading to non-traumatic amputation. According to the International Diabetes Federation, DFU-related amputations account for 84% of non-traumatic lower limb amputations worldwide, with a mortality rate of more than 70% 5 years after amputation (13). Therefore, it is essential to improve the management of DFU with limb salvage therapy (14). Alternating positive and negative pressure is beneficial to promote the constriction of small blood vessels around the wound and enhance tissue perfusion. Negative pressure vacuum cupping therapy increases the blood perfusion of the wound, promotes the mechanical traction of the wound edge, and induces angiogenesis through intermittent and sufficient negative pressure drainage (15). At the same time, the mechanical stress induced by negative pressure vacuum cupping therapy promotes the proliferation of fibroblasts and the expression of vascular endothelial growth factor, enhances collagen deposition and neovascularization, removes wound secretions, and increases granulation tissue formation. Finally, the negative pressure environment physically limits bacterial invasion, thereby reducing tissue edema, optimizing cell migration and re-epithelialization, and improving the local microenvironment, which ultimately promotes wound healing (16). The equipment used is a medical-grade negative pressure vacuum cupping device, typically consisting of a simple plastic cup equipped with a manual air pump. A high level of negative pressure is rapidly generated through the manual air pump and subsequently maintained passively. During the procedure, the rim of the cup is placed directly on intact or nearly intact skin, forming an open interface between the skin surface and the internal space of the cup. This method does not involve filling the wound bed or utilizing specialized dressings; instead, it relies solely on the adhesion of the cup’s rim to the skin to achieve basic physical adsorption and sealing. Cupping is performed after wound cleaning during dressing changes. The frequency and intensity of suction applied during a single dressing session are determined based on the assessment of wound exudate levels. Upon completion of the cupping process, the wound is cleaned again, followed by the application of a moist dressing. Depending on the exudate condition, either foam dressings or standard medical dressings are used to ensure proper sealing. This approach is straightforward, easy to implement, and cost-effective. During the treatment period, dressing changes are initially conducted on a daily basis. Subsequently, the frequency is adjusted according to the wound status and exudate volume. The total number of dressing changes does not exceed 30, and the overall treatment cost remains within 3,000 RMB (approximately 418 $).

Wet healing aims to significantly accelerate wound repair by maintaining the moderately moist physiological environment of the wound. Firstly, in this study, it promoted the migration and proliferation of epithelial cells, and the wet environment prevented the formation of dry scab and removed the physical barriers, thereby enhancing the migration rate of epidermal cells by more than 40%. At the same time, wet healing can stimulate the release of vascular endothelial growth factor and epidermal growth factor to accelerate angiogenesis and collagen synthesis. Growth factors are usually downregulated in the high glucose environment of diabetic foot; therefore, wet healing should be combined with silver ion dressing or growth factor gel (17). Secondly, wet healing can activate autolytic debridement and enzymatic activity. Matrix metalloproteinases in wound exudates maintain high activity under humid conditions, dissolve necrotic tissues and fibrin, and achieve painless debridement. Compared to the dry environment, the wet state can retain and enhance the degradation of bioactive substances. The closed dressing formed a low oxygen-tension environment, which promoted angiogenesis and fibroblast proliferation. Constant temperature and humidity can reduce nervous stimulation, alleviate pain, block bacterial invasion, and reduce the infection rate to 2.6% (18).

During the treatment process, attention should be paid to systemic treatment, blood glucose and blood pressure control, anti-biotic therapy, circulatory improvement, nutritional support, and maintenance of electrolyte balance. Adjunctive antibiotics (ciprofloxacin 500 mg bid for E. coli control) may improve the efficacy of Negative pressure vacuum cupping, while nutritional support (20 g/day whey protein isolate) can significantly increase the serum levels of prealbumin. At the same time, standardized local wound dressing change should be strictly implemented to control infection and promote healing (19). The medical team should adopt effective psychological interventions, determine accessible goals, enhance patients’ confidence in overcoming the disease, relieve patients’ anxiety, and improve patients’ compliance.

## 7 Conclusion

This case highlights the significant benefits of comprehensive care for diabetic foot, integrating traditional and modern therapies. The combination of negative pressure vacuum cupping with wet therapy increased wound closure rates, improved circulation with the establishment of collateral circulation, and significantly reduced the risk of limb amputation. Importantly, this integrated approach not only accelerated healing but also significantly improved the quality of life of the patient. This experience provides crucial evidence for updating clinical practice guidelines and establishing a valuable reference for optimizing diabetic foot. This report underscores the necessity for future research and development based on multidisciplinary strategies to improve patients’ outcomes.

## 8 Limitations

Severe diabetic foot ulcers can lead to progressive tissue necrosis, and severe refractory osteomyelitis can lead to sepsis complicated with multiple organ failure. Most of the clinical methods are amputation, but amputation is not the only outcome for patients. The exploratory use of negative pressure cupping to improve the peripheral circulation of patients in the treatment of diabetic foot requires the full trust of patients in the wound therapist before it can be implemented. While this case report illustrates the efficacy of wet healing and cupping wet healing, it is important to acknowledge the limited sample size and the need for future randomized controlled trials to confirm the clinical application value of this treatment strategy. Meanwhile, when negative-pressure cupping therapy is clinically employed, in the case of diabetic foot complicated by neuropathy or ischemia, it is likely to cause skin damage and severe contusions, and may even exacerbate ulcers or infections. Thus, it must be used with prudence under professional guidance. Follow-up studies should assess the effect of different levels of negative pressure on wound healing and explore the feasibility of other integrated approaches. As a single-case study, causal attribution remains limited.

## Data Availability

The raw data supporting the conclusions of this article will be made available by the authors, without undue reservation.
